# Structures of disease-specific serum alpha-fetoprotein isoforms

**DOI:** 10.1054/bjoc.2000.1441

**Published:** 2000-10-26

**Authors:** P J Johnson, T C W Poon, N M Hjelm, C S Ho, C Blake, S K W Ho

**Affiliations:** Department of Clinical OncologyDepartment of Chemical Pathology at the Sir YK Pao Centre for Cancer, Chinese University of Hong Kong, Shatin, Hong Kong

**Keywords:** hepatocellular carcinoma, non-seminomatous germ cell tumours, alpha-fetoprotein isoform, protein structure, glycosylation

## Abstract

Alpha-fetoprotein (AFP) is widely used as a serological marker in the diagnosis of hepatocellular carcinoma (HCC) and non-seminomatous germ cell tumours (NSGCT). By application of isoelectric focusing (IEF) disease-specific AFP isoforms can be identified. Three major bands are apparent: + l (associated with ‘benign’ liver disease), + II (associated with HCC) and +III (associated with NSGCT). Recently, we have characterized the predominant glycans of human serum AFP and now report the application of these findings and electrospray ionization-mass spectrometry (ESI-MS) to the determination of the glycan composition of the isoforms present in the sera of 12 patients with HCC and of one patient with NSGCT. ESI-MS allowed simultaneous identification of various AFP glycoforms in purified serum AFP. Seven glycoforms were identified, but with different abundance in the sera of the HCC patients, whereas six glycoforms were identified in the serum from the NSGCT patient. The glycan structures of these glycoforms were deduced from their observed masses. AFP glycoforms carrying a single biantennary complex-type *N*-glycan appeared as the predominant glycoforms, whereas those carrying both *N*-glycan and *O*-glycan appeared as minor glycoforms. Correlation between the abundance of the AFP glycoforms and the IEF band intensity suggested that different degrees in sialylation cause the formation of isoforms. This contention was subsequently supported by the ESI-MS and kinetic in vitro desialylation studies on purified Bands + l and + lI AFPs. Our findings indicate that HCC-associated isoforms (Band + II) represent a group of glycoproteins whose carbohydrate structures are all characterized by being mono-sialylated, whereas those associated with benign liver disease and NSGCT are di- and a-sialo species, respectively. Knowledge of the structure of the tumour-specific isoforms should form an important basis for clinically useful assays. © 2000 Cancer ResearchCampaign


*British Journal of Cancer* (2000) **83**(10), 1330–1337
© 2000 Cancer Research Campaign
doi: 10.1054/ bjoc.2000.1441, available online at http://www.idealibrary.com on IDEAL®

### Structures of disease-specific serum alpha-fetoprotein isoforms

**PJ Johnson[1], TCW Poon[1], NM Hjelm[2], CS Ho[2], C Blake[1] and SKW Ho[1]**

[1]Department of Clinical Oncology and [2]Department of Chemical Pathology at the Sir YK Pao Centre for Cancer, Chinese University of Hong Kong, Shatin, Hong Kong

**Summary** Alpha-fetoprotein (AFP) is widely used as a serological marker in the diagnosis of hepatocellular carcinoma (HCC) and non-seminomatous germ cell tumours (NSGCT). By application of isoelectric focusing (IEF) disease-specific AFP isoforms can be identified. Three major bands are apparent: + I (associated with 'benign' liver disease), + II (associated with HCC) and +III (associated with NSGCT). Recently, we have characterized the predominant glycans of human serum AFP and now report the application of these findings and electrospray ionization-mass spectrometry (ESI-MS) to the determination of the glycan composition of the isoforms present in the sera of 12 patients with HCC and of one patient with NSGCT. ESI-MS allowed simultaneous identification of various AFP glycoforms in purified serum AFP. Seven glycoforms were identified, but with different abundance in the sera of the HCC patients, whereas six glycoforms were identified in the serum from the NSGCT patient. The glycan structures of these glycoforms were deduced from their observed masses. AFP glycoforms carrying a single biantennary complex-type *N*-glycan appeared as the predominant glycoforms, whereas those carrying both *N*-glycan and *O*-glycan appeared as minor glycoforms. Correlation between the abundance of the AFP glycoforms and the IEF band intensity suggested that different degrees in sialylation cause the formation of isoforms. This contention was subsequently supported by the ESI-MS and kinetic in vitro desialylation studies on purified Bands + I and + II AFPs. Our findings indicate that HCC-associated isoforms (Band + II) represent a group of glycoproteins whose carbohydrate structures are all characterized by being mono-sialylated, whereas those associated with benign liver disease and NSGCT are di- and a-sialo species, respectively. Knowledge of the structure of the tumour-specific isoforms should form an important basis for clinically useful assays. © 2000 Cancer Research Campaign

**Keywords:** hepatocellular carcinoma; non-seminomatous germ cell tumours; alpha-fetoprotein isoform; protein structure; glycosylation

Measurement of serum alpha-fetoprotein (AFP, reference range < 10 ng ml$^{-1}$) provides a marker for the diagnosis and management of hepatocellular carcinoma (HCC) (Johnson et al, 1978; Nomura et al, 1985; Sheu et al, 1985) and non-seminomatous germ cell tumours (NSGCT) (Lange and Fralay, 1977; Javadpour 1980; Kay et al, 1987; Nichols et al, 1990). About 70% of patients with HCC will have levels above the reference range (Johnson et al, 1978; Nomura et al, 1985; Sheu et al, 1985). A serum concentration of greater than 500 ng ml$^{-1}$, in an HCC high-incidence area, and in the appropriate clinical setting, is usually diagnostic of HCC. However, modestly raised levels of AFP (10–500 ng ml$^{-1}$) are also common in non-malignant chronic liver disease, so that the specificity of the AFP test for HCC tends to be low (Johnson et al, 1978; Okuda, 1986; Lok and Lai, 1989). This represents a serious clinical drawback for the test since most cases of HCC arise in patients with concurrent chronic liver disease (Kew and Popper, 1984; Johnson and Williams, 1987).

AFP is a glycoprotein consisting of 591 amino acids that has been reported to have a single asparagine linked complex-type sugar chain (Tarelli et al, 1992; Ferranti et al, 1995). Although it has been known for over 20 years that serum AFP exhibits micro-heterogeneity when examined by isoelectric focusing (IEF), the biomolecular basis for this heterogeneity has remained controversial (Ruoslahti 1979; Smith and Kelleher, 1980). Recently, we have used fluorescence labeling, sequential exoglycosidase digestion, high-performance liquid chromatography and matrix-assisted laser desorption ionization in time-of-flight mass spectrometry, to determine the glycan structures of purified serum AFP from patients with HCC and NSGCT. Surprisingly, eleven major glycans were found, of which seven were *N*-linked, and four were *O*-linked, to the protein backbone (Figure 1) (Johnson et al, 1999).

Several attempts have been made to identify an 'HCC-specific' glycoform, aiming thereby to improve the specificity of AFP as a diagnostic test for HCC. The most successful approach has been based on the difference in the binding affinity of the AFP glycoforms to various lectins, particularly lentil lectin and Concanavalin A. Indeed, different binding patterns were found in AFP from patients with different diseases (Smith and Kelleher, 1980; Buamah et al, 1986; Du et al, 1991). More recently, using IEF to directly identify isoforms of AFP, the Band + II and Band + III AFP isoforms were shown to be highly specific for HCC (Burditt et al, 1994; Ho et al, 1996) and NSGCT (Johnson et al, 1995), respectively. A unique IEF pattern is also found for the AFP that arises in patients with chronic liver disease without any evidence of malignancy (Ho et al, 1996; Johnson et al, 1997). Preliminary studies have suggested that screening for the Band + II isoform may allow early, even preclinical, diagnosis of HCC in high-risk patients (Johnson et al, 1997). However, the current protocol of IEF and immunoblotting techniques for identification of the tumour-specific isoform is very time-consuming and it is therefore

*Received 18 February 2000*
*Revised 10 July 2000*
*Accepted 11 July 2000*

*Correspondence to:* PJ Johnson

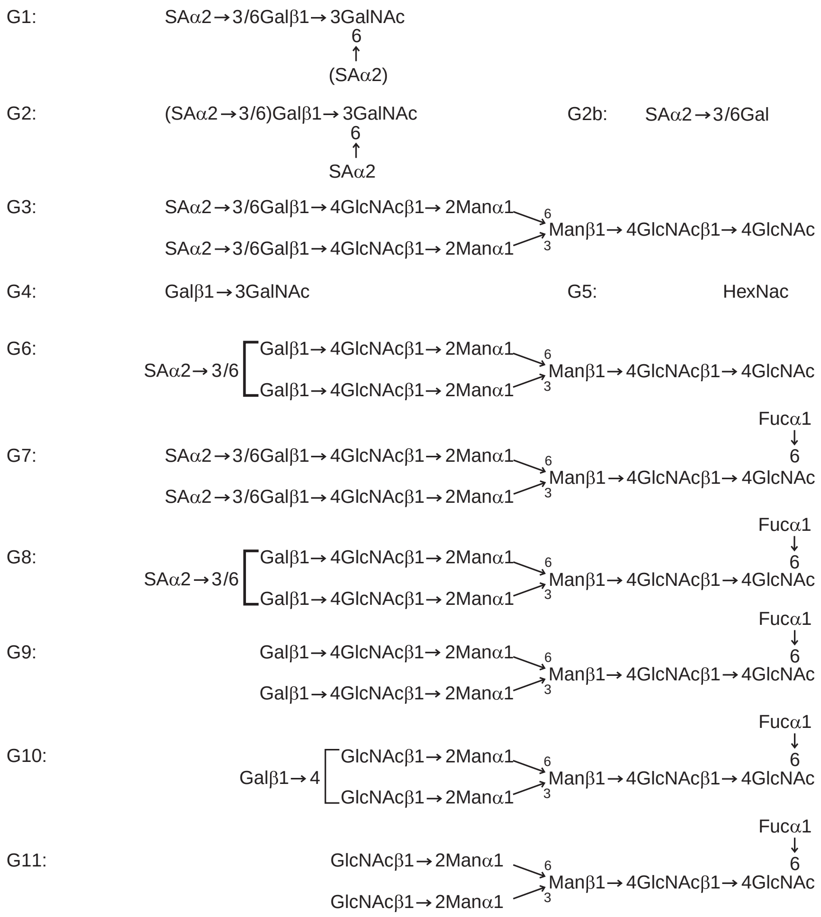

**Figure 1** The structures of the glycans on AFP associated with HCC and NSGCT

important to develop a simpler and more rapid assay. To achieve this, it is essential to determine the biomolecular basis of the disease-specific isoforms so as to identify any distinctive structural features.

Although the carbohydrate structures of serum disease-specific glycoforms have been proposed using the lectin affinity electrophoretic technique (Shimizu et al, 1996), the structures of the HCC and NSGCT isoforms identified by IEF remain unknown. In this study, using electrospray ionization-mass spectrometry (ESI-MS) and applying our recent characterization of the 11 glycans of AFP, we have elucidated the structures of the glycan moieties of those AFP isoforms associated with disease-specific states, and related these to the IEF banding patterns. ESI-MS and kinetic in vitro desialylation studies were then applied to confirm the structures of disease-specific isoforms.

### MATERIALS AND METHODS

### Patient sera

Sera were collected from 12 patients with histologically confirmed HCC, all of whom had serum AFP levels of > 300 000 ng ml$^{-1}$ (range 300 000–300 000 ng ml$^{-1}$), and one patient with histologically confirmed NSGCT with a serum AFP level of 17 820 ng ml$^{-1}$. The concentration of serum AFP was assayed by a microparticle enzyme immunoassay (MEIA, Abbott Laboratories, Illinois, USA). Sera were stored at –70°C until further analysis, or until purification of the AFP.

### Purification of AFP by one-step affinity chromatography

Rabbit anti-human AFP (DAKO, Glostrup, Denmark) was coupled to CNBr-activated Sepharose 4B (Pharmacia Biotech, Uppsala, Sweden). A mini-column containing 1 ml of coupled gel was equilibrated with phosphate buffered saline (PBS, 10 mM sodium phosphate, 10 mM KCl, 127 mM NaCl, pH 7.4). The sample to be purified (200–500 µg) was loaded into the column and incubated at room temperature for 4 h. The column was then washed with 50 ml of PBS containing 0.2% Tween 20, and subsequently with 50 ml of PBS. Bound AFP was eluted with 20 ml of 0.2 M $Na_2CO_3$ under gravity. The 'total' AFP concentration was determined as previously described. The purity of the final AFP preparation was regarded as acceptable when a single band, with a molecular weight of about 68 kDa, was visualized on an SDS PAGE gel using silver staining.

© 2000 Cancer Research Campaign

### Purification of AFP isoforms by preparative IEF and affinity chromatography

Serum containing about 5 mg of AFP was applied to a mixture of 5% sorbitol, 10% glycerol and ampholytes (6.25% Pharmalyte pH 4.5–5.4, Pharmacia Biotech) which had been pre-focused at constant power (15 W) for 30 min in a Rotofor cell (Biorad, California, USA) at 4°C. The sample was then focused at constant power (15 W) until the voltage stabilized and then focused for a further 30 min before 20 individual 2 ml fractions were harvested. pH and AFP concentration in each fraction were measured. Fractions with more than 10 μg of AFP were pooled and refractionated to improve the separation of the isoforms. The pH and AFP concentration were again measured. Fractions containing only Band +I or Band +II AFP, free of other isoforms, were identified by gel IEF. Finally, the separated AFP isoforms in the pooled fractions were purified by affinity chromatography.

### Isoelectric focusing-immunoblotting (IEF-IB)

The method of Burditt et al (1994) with minor modifications was used. Samples were focused in 1.5 mm thick agarose gel containing 1% agarose (IEF grade Type VIII, Sigma, Missouri, USA) 5% sorbitol, 10% glycerol and ampholytes (6.25% Pharmalyte pH 4.5–5.4, Pharmacia Biotech) at 10°C. After pre-focusing at 1500 V for 30 min, samples were then applied directly to the gel and focused at 2000 V for 1 h. The focused proteins were transferred to nitrocellulose membrane, and then incubated with horseradish peroxidase conjugated polyclonal rabbit anti-human AFP (DAKO). After washing, the enhanced chemiluminescence detection system (ECL, Pharmacia Biotech) was used to visualize the AFP protein bands.

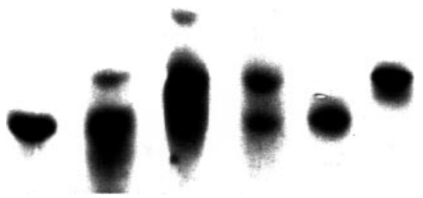

**Figure 2** Typical IEF patterns of serum AFPs from patients with liver cirrhosis only (LC), HCC (H) and NSGCT (G), and purified total AFP (T), Band +I AFP (+I) and Band +II AFP (+II)

### ESI-MS analysis of purified AFPs

ESI-MS was performed on a triple quadrupole Quattro II system (Micromass, Manchester, UK). The mass scale was calibrated with a mixture of myoglobin and trypsinogen. The capillary voltage of the electrospray probe was set to + 3.5 KV. The nebulizer gas was set at 20 l $h^{-1}$ and the drying gas at 250 l $h^{-1}$. The source temperature was set at 75°C. The desalted AFP sample (5 pmol $\mu l^{-1}$ in 50% acetonitrile in water containing 0.2% formic acid) was injected into the ion source at a flow rate of 5 μl $min^{-1}$. Positive ionization mass spectra were acquired over the mass/charge ratio (M/Z) range of 1000–2400 at 4 s per scan. The sampling cone voltage was varied through the range of 47–90 V. The background subtracted raw data was processed by the Maximum Entropy (MaxEnt) software to produce the transformed mass spectrum.

**Table 1** The predominant AFP glycoforms in HCC identified by ESI-MS, and their deduced glycan compositions. Purified total AFPs from the sera of HCC patients were subjected to ESI-MS analysis. All samples were measured for at least four times. The observed masses of the identified AFP glycoforms were matched with the calculated masses of the hypothetical AFP glycoforms. Those observed AFP glycoforms in which mass difference was less than 0.01% were considered to have the same molecular structure as the matched hypothetical AFP glycoforms

| Observed mass | Deduced glycan composition | Calculated mass | Monosaccharide composition per protein molecule | Label |
|---|---|---|---|---|
| 69048.5 ± 3.6 | G11 + G1 + G5 | 69044.3 | 2 SA, 4 Hex,<br>6 HexNAc,<br>1 DeoxyHex | +Ia |
| 68797.4 ± 3.4 | G7[a]<br>G11 + G2b + G2b | 68800.0 | 2 SA, 5 Hex,<br>4 HexNAc,<br>1 DeoxyHex | +Ib |
| 68651.2 ± 3.8 | G3 | 68653.9 | 2 SA, 5 Hex,<br>4 HexNAc | +Ic |
| 68754.4 ± 9.7 | G11 + G2 + G5 | 68753.1 | 1 SA, 4 Hex,<br>6 HexNAc,<br>1 DeoxyHex | +IIa |
| 68708.0 ± 8.5 | G8 + G5[a]<br>G10 + G2<br>G10 + G2b + G5<br>G11 + G2b + G4 | 68712.0<br>68712.0<br>68712.0<br>68712.0 | 1 SA, 5 Hex,<br>5 HexNAc,<br>1 DeoxyHex | +IIb |
| 68505.8 ± 3.1 | G8 | 68508.8 | 1 SA, 5 Hex,<br>4 HexNAc,<br>1 DeoxyHex | +IIc |
| 68358.7 ± 4.6 | G6 | 68362.7 | 1 SA, 5 Hex,<br>4 HexNAc | +IId |

[a]Most probable glycan composition within the group

© 2000 Cancer Research Campaign

**Table 2** The predominant AFP glycoforms in NSGCT identified by ESI-MS, and their deduced glycan compositions. Purified total AFP from the serum of a NSGCT patient (as per Table 1)

| Observed mass | Deduced glycan composition | Calculated mass | Monosaccharide composition per protein molecule | Label |
|---|---|---|---|---|
| 68803 ± 1.5 | G7<br>G11 + G2b + G2b | 68800.5 | 2 SA, 5 Hex,<br>4 HexNAc,<br>1 DeoxyHex | +Ib |
| 68712 ± 0.8[a] | G10 + G2<br>G10 + G2b + G5<br>G11 + G2b + G4<br>G8 + G5 | 68712.0<br>68712.0<br>68712.0<br>68712.0 | 1 SA, 5 Hex,<br>5 HexNAc,<br>1 DeoxyHex | +IIb |
| 68511 ± 1.6 | G8 | 68508.8 | 1 SA, 5 Hex,<br>4 HexNAc,<br>1 DeoxyHex | +IIc |
| 68342 + 2.3 | G11 + G2b | 68346.7 | 1 SA, 4 Hex,<br>4 HexNAc,<br>1 DeoxyHex | +IIe |
| 68423 ± 0.6[c] | G10 + G4[d]<br>G9 + G5 | 68420.7<br>68420.7 | 5 Hex, 5 HexNAc,<br>1 DeoxyHex | +IIIa |
| 68053 ± 1.1[b] | G10 | 68055.4 | 4 Hex, 4 HexNAc,<br>1 DeoxyHex | +IIIb |

[a]First predominant glycoform; [b]second predominant glycoform; [c]third predominant glycoform; [d]most probable glycan composition within the group

**Table 3** Distribution of the major AFP glycoforms in 12 HCC patients. Total AFPs were purified from individual HCC patient serum by affinity chromatography, and subjected to ESI-MS analysis. The glycan compositions of the identified glycoforms were deduced from their observed masses

| HCC case | 1 | 2 | 3 | 4 | 5 | 6 | 7 | 8 | 9 | 10 | 11 | 12 |
|---|---|---|---|---|---|---|---|---|---|---|---|---|
| | | | | | | | +Ia | | +Ia | +Ia | | |
| AFP | +Ib[a] | +Ib[a] | +Ib[b] | +Ib | +Ib | +Ib[a] | +Ib[a] | +Ib | +Ib[a] | +Ib[a] | +Ib[a] | +Ib[a] |
| Glycoforms | +Ic | | +Ic[a] | +Ic[a] | +Ic[a] | +Ic | | +Ic[a] | | +Ic | +Ic[b] | +Ic |
| | | | | | +IIa | | | | +IIa | | | |
| | | | | +IIb | +IIb | | | | | | | |
| | +IIc[b] | +IIc[b] | +IIc | | | +IIc[b] | +IIc[b] | +IIc | +IIc[b] | +IIc[b] | | +IIc |
| | | | +IId | +IId[b] | +IId[b] | +IId | | +IId[b] | | +IId | +IId | +IId[b] |

[a]Predominant AFP glycoform; [b]second predominant AFP glycoform

The accuracy of this method is ± 0.01%. All samples were measured for 3–5 times.

### Deduction of the glycan composition in AFP glycoforms identified by ESI-MS

With the knowledge of the structures of eleven predominant glycans on AFP glycoforms, the molecular masses for a series of hypothetical AFP glycoforms comprising random combinations of the *N*-linked and/or *O*-linked glycans were calculated. Their theoretical masses (average masses) were derived from the theoretical mass of the AFP protein core (calculated with the Peptide Mass software from ExPASy, accessed through the Internet at http://expasy.hcuge.ch), and the theoretical masses of the monosaccharide residues reported by Dell et al (1993). The observed masses of the AFP glycoforms identified by ESI-MS were then matched with the calculated masses of the hypothetical AFP glycoforms. Those observed AFP glycoforms in which mass difference was less than 0.01% were considered to have the same molecular structure as the matched hypothetical AFP glycoforms.

### Kinetic in vitro desialylation of purified Band + I AFP

20 μl of 250 mU $ml^{-1}$ sialidase (*Vibrio cholerae* neuraminidase, Roche Diagnostics, Indianapolis, USA) was mixed with 10 μl of purified Band + I AFP (800 μg $ml^{-1}$) in reaction buffer (0.1 M Na acetate, 9 mg $ml^{-1}$ NaCl, 1 mg $ml^{-1}$ $CaCl_2$, pH 5.6), and incubated at 37°C. At the assigned time for the individual assay tubes, the reaction was stopped by adding 20 μl of stopping buffer (0.15 M glycine-NaOH, pH 11.0). The final reaction mixture was subjected to IEF-IB for the visualization of the AFP IEF banding patterns.

### RESULTS

### Identification of glycan composition of AFP glycoforms in patient sera by ESI-MS

Total AFP was successfully purified from individual patient sera (Figure 2), and subjected to ESI-MS analysis. Four major and three minor AFP glycoforms, purified from the sera of the HCC patients, were identified (Table 1). Three major and three minor

© 2000 Cancer Research Campaign 

AFP glycoforms were identified in the serum from the NSGCT patient (Table 2). Their compositions were deduced from the observed molecular masses after being matched with the hypothetical AFP glycoforms as described above. In total, 10 AFP glycoforms were identified from the patient sera. For seven out of the 10 identified AFP glycoforms, there was only one possible glycan composition that could be deduced from the observed mass for each of the glycoforms.

Although three out of 10 identified AFP glycoforms (AFPs + Ib, + IIb and + IIIa) were matched with more than one hypothetical AFP glycoforms (Tables 1 and 2), the comparison with those identified glycoforms with concrete glycan moiety(s) can provide a clue to decide which one is the most probable glycoform. In the case of AFP + Ib, its expected theoretical mass is greater than that of AFP + Ic by 146.1, which is equivalent to the mass of a fucose. It is possible that AFP + Ib has a glycan moiety similar to AFP + Ic, but with an additional fucose. Such postulation helps to explain why only either AFP + Ib (67%, 8 of 12) or AFP + Ic (33%, 4 of 12) appeared as the predominant glycoforms in sera from HCC patients (Table 3). The presence of AFP + Ib as the predominant glycoform may be caused by a higher fucosylation activity in hepatoma cells. Therefore, the fucosylated disialo biantennary complex-type *N*-linked glycan (G7) is the most probable glycan moiety of the AFP + Ib, which is consistent with the identification of G7 glycans as one of the major glycans released from HCC-associated AFP (Yoshima et al, 1980; Aoyagi et al, 1993; Johnson et al, 1999). In the case of AFP + IIb, it was only found in sera with a negligible amount of AFP + IIc (Table 3). The expected theoretical mass of AFP + IIb is greater than that of AFP + IIc by 203.2, which is equivalent to the mass of an HexNAc residue (G5). The absence of the AFP + IIc could be possibly explained by the addition of a G5 glycan to AFP + IIc, converting it to AFP + IIb. Therefore the most probable glycan moieties of AFP + IIb are G8 and G5 glycans. For the AFP glycoforms identified in the NSGCT serum, because G10 glycan is the sole contributor of the carbohydrate content of the second predominant glycoform, + IIIb, it is reasonable to postulate that the AFP + IIIa also carried one G10 glycan, but with an additional G4 glycan (Table 2). Thus, it appears that glycoforms carrying a G10 glycan predominated in the NSGCT-derived AFP, and contributed to glycoforms + IIb, + IIIa and + IIIb.

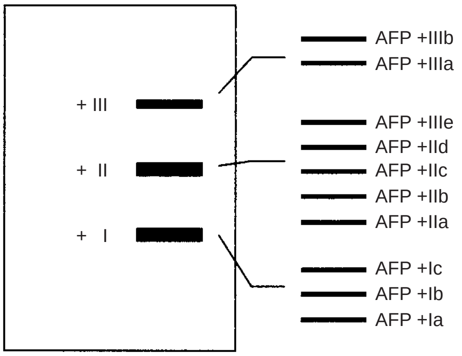

**Figure 3** Diagrammatic presentation of the glycoforms comprising the AFP IEF bands +I, +II and +III. The IEF banding pattern of AFP glycoforms is postulated to be dictated mainly by the degree of sialylation on the glycan chain(s) of the protein. This is confirmed by the ESI-MS analysis and the kinetic in vitro desialylation study of the purified AFP isoforms. Class +I, +II and +II AFPs will be focused as Band +I, +II and +III, respectively

### Classification of the identified AFP glycoforms

Since it is known that the isoelectric point (pI value) a glycoprotein is affected by the number of sialic acid residues (Stibler, 1991), the identified AFP glycoforms were classified according to their sialic acid content – Class + I, Class + II and Class + III, carrying two, one and no sialic acid residues, respectively. In Class + I there were three subtypes (+ Ia–+Ic); in Class + II there were five subtypes (+ IIa–+IIe); in Class + III there were two subtypes (+ IIIa and + IIIb). The classification of the identified AFP glycoforms from sera of the HCC patients and from the NSGCT patient are summarized in

**Table 4** The major serum AFP glycoforms, identified by ESI-MS, in AFP preparations that were purified from two HCC patients. Total AFP, Band +I AFP and Band +II AFP were purified by affinity chromatography and preparative IEF, and subsequently subjected to ESI-MS analysis for 3–5 determinations. The glycan compositions of the identified glycoforms were deduced from their observed massess

| | Observed mass by ESI-MS | | | Identity (calculated mass) | Deduced glycan composition |
|---|---|---|---|---|---|
| | Total AFP | Band +I AFP | Band +II AFP | | |
| Patient 1 | 68361[a] | – | 68362 | AFP +IId (68362.7) | G6 |
| | 68655 | 68648 | – | AFP +Ic (68653.9) | G3 |
| | 68801 | 68797 | – | AFP +Ib (68800.0) | G7 |
| Patient 2 | 68352 | – | 68355 | AFP +IId (68362.7) | G6 |
| | 68507 | – | 68505 | AFP +IIc (68506.3) | G8 |
| | 68651 | 68655 | – | AFP +Ic (68653.9) | G3 |
| | 68797 | 68802 | – | AFP +Ib (68800.0) | G7 |

[a]appearing as a minor glycoform

*© 2000 Cancer Research Campaign*

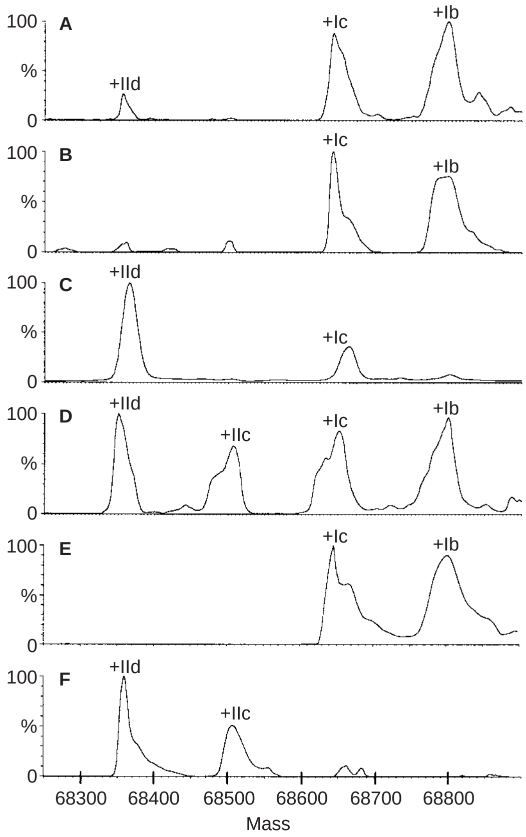

**Figure 4** ESI mass spectra of the AFP glycoforms in the total AFP (**A, D**), Band +I AFP (**B, E**) and Band +II AFP (**C, F**) preparations. AFPs were purified from the sera of two HCC patients (Patient 1: **A, B, C**; Patient 2: **D, E, F**) by affinity chromatography. Band +I and +II AFPs were separated by preparative IEF before purification

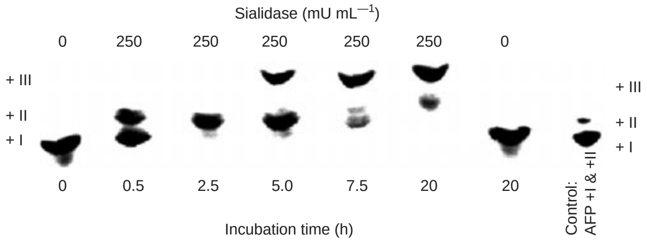

**Figure 5** Conversion of Band +I AFP to Band +II and +III AFPs by sialidase digestion. Band +I AFP was purified by preparative IEF and affinity chromatography, and digested with sialidase. The digestion products were monitored at different time points by IEF-IB. Consistent findings were observed on two independent occasions

Tables 1 and 2, respectively. After grouping according to the sialic acid content, it was apparent that all the identified glycoforms had similar carbohydrate contents (9–11 neutral monosaccharide residues). The similarity in the protein core and amount of neutral monosaccharides in all the identified glycoforms strongly suggest that differences in the pl values of the glycoforms are dictated mainly by the number of sialic acid residues.

### Distribution and abundance of the identified AFP glycoforms in different patients

The distribution of the AFP glycoforms in the sera of patients with HCC is presented in Table 3. The pattern is heterogeneous but several consistent features emerge. In all cases Class + I AFPs predominated, whereas Class + II AFPs appeared as the second predominant glycoforms. AFP glycoforms carrying a single disialylated biantennary complex-type *N*-glycan (G3 or G7) predominated in Class + I AFPs, whereas AFP glycoforms carrying a single monosialylated biantennary complex-type *N*-glycan (G6 or G8) predominated in Class + II AFPs. AFP glycoforms carrying both the *N*-glycan and *O*-glycan appeared as minor glycoforms in the patient sera.

Owing to the rarity of NSGCT patient serum samples with sufficiently high levels of AFP for analysis, we only have been able to use ESI-MS to elucidate the structure of AFP glycoforms derived from a single case of NSGCT. No claim, therefore, has been made for the predominant glycoforms of NSGCT-associated AFP. However, AFP glycoforms carrying an asialylated biantennnary complex-type *N*-glycan (G10) appeared as the predominant glycoforms in Class + III AFPs.

### Deduction of glycan composition of AFP isoforms identified by IEF

In all serum samples from the HCC patients, comparison of the intensities of Band + I and Band + II showed that Band + I predominated (Figure 2). Similarly, ESI-MS identified Class + I AFPs as the predominant glycoforms (Table 3). By matching the abundance, we postulate that Class + I and Class + II AFPs represent Band + I and Band + II on the IEF gel, respectively (Figure 2 and Table 3). Similarly in the case of NSGCT, Class + II and + III AFPs should represent Bands + II and + III, respectively (Figure 2 and Table 2).

As the addition of one sialic acid residue decreases the pl value (of a glycoprotein) by about 0.1 pH (Stibler, 1991), Class + I AFPs should, if our postulation is correct, have a pl value lower than that of Class + II AFPs by about 0.1 pH, and the pl value of Class + II AFPs lower than that of Class + III AFPs, again by about 0.1 pH. Consistent with this prediction, the pl values of the IEF bands were: Band + I = pI 4.78, Band + II = pl 4.83 and Band + III = pI 4.90 (Figure 2). The proposed distribution of Class + I, Class + II and Class + III AFPs on the gel IEF pattern is summarized in Figure 3. It is apparent that more than one subtype of each class of AFPs usually coexisted in individual patient serum (Tables 2 and 3). Furthermore, thick bands were usually seen on the IEF gel (Figure 2). We suggest that, as summarized in Figure 3, several AFP isoforms, all with very close pl values (those within a class), are focused at similar positions. Their bands overlap with each other on the IEF gel, and finally appear as a single thick band.

### Identification of glycan compositions of purified Band + I and Band + II AFPs by ESI-MS

To further support our postulated structures for the specific IEF bands, besides the total AFP we prepared purified Band + I and + II AFPs from two HCC patients (Figure 2), and subjected them to ESI-MS analysis. Unfortunately, the level of Band + III AFP in the NSGCT serum was too low to allow us to purify a sufficient

© 2000 Cancer Research Campaign amount for ESI-MS analysis. By ESI-MS (Figure 4 and Table 4), both Class +I (putatively disialylated) and Class +II (putatively monosialylated) AFPs were identified in the purified total AFP preparations (comprising all the AFP glycoforms existing in the sera). As predicted, Class +I (disialylated) AFPs were, indeed, predominantly retained in the Band +I AFP preparations, whereas the Class +II (monosialylated) AFPs were predominantly retained in the Band +II AFP preparations. However, fucosylated and non-fucosylated AFP glycoforms were not differentially separated by preparative IEF. The results of these ESI-MS experiments indicate that Class +I (disialylated) and Class +II (monosialylated) AFPs were focused as Band +I and +II AFPs, respectively.

### Kinetic in vitro desialylation of purified Band +I AFP

In the presence of sialidase, the purified Band +I AFP, in a step-wise manner, migrated from the position +I to +II and finally to +III (Figure 5). Consistent findings were observed on two independent occasions. Upon partial sialidase digestion (0.5 h of digestion), microheterogeneity increased immediately, showing a new alkaline band at position +II. After 2.5 h of digestion, most of Band +I AFP was converted to the glycoform at position +II. After 5 h of digestion, another glycoform at position +III appeared. Upon complete digestion (20 h of digestion), the majority of the AFP protein molecules (asialylated AFP) were still focused as Band +III. Non-specific effects of incubation conditions were excluded by control experiments. Conversion of Band +I AFP to Band +II and +III AFP upon sequential removal of sialic acid residues confirmed that the appearance of alkaline AFP glycoforms (+II and +III) identified by IEF were attributable to a lower sialic acid content, and asialylated AFP was focused as Band +III. Thus, this kinetic in vitro desialylation experiment strongly supports our contention that Band +I, +II and +III are composed of di-, mono- and a-sialylated AFPs, respectively.

### DISCUSSION

Although AFP has been used as a tumour marker for the diagnosis of HCC for many years, its specificity is poor when levels fall within the ‘grey area’, i.e. 10–500 ng ml$^{-1}$ (Johnson et al, 1978; Okuda, 1986; Lok and Lai, 1989). In this range, differentiation between benign and malignant liver diseases cannot be made with confidence on the basis of serum AFP levels alone. There is thus a need to find a method of increasing the specificity of AFP. Lentil lectin-reactive AFP has previously been proposed as a diagnostic marker to discriminate between AFP arising from HCC and AFP arising from benign liver disease (Du et al, 1991; Taketa et al, 1993; Shimizu et al, 1996). Using the lectin binding properties of *N*-glycans with known structures (Yamashita et al, 1993) or glycosidase digestion (Shimizu et al, 1996) together with lectin affinity electrophoresis, the carbohydrate structure of the major HCC-specific AFP glycoform has been suggested indirectly to be of the fucosylated monosialo biantennary complex-type.

Identification of AFP isoforms is an alternative method to increase the specificity of AFP. Using IEF and immunoblotting, AFP isoforms appearing as Band +II and Band +III were found to be relatively specific for HCC and NSGCT, respectively (Johnson et al, 1995; Ho et al, 1996; Johnson et al, 1997). The direct structural analysis of these AFP isoforms in individual patients has, until recently, been hindered by the extremely small amounts of serum AFP available. However, with the help of ESI-MS and a random assembly model (i.e. the construction of hypothetical AFP glycoforms from the AFP protein core and the known glycans identified on AFP), we report here an approach that has permitted simultaneous identification of the predominant AFP glycoforms in individual patients. The present study provides evidence that the glycosylation of human serum AFP is more complex than the single biantennary complex-type *N*-glycan that has previously been reported. There exist AFP glycoforms carrying both *O*-glycan and *N*-glycan. The identification of AFP glycoforms carrying *O*-glycan was consistent with our previous identification of *O*-glycans released from AFP molecules (Johnson et al, 1999).

Our results show that an AFP glycoform carrying a single monosialo biantennary complex-type *N*-glycan, either fucosylated or not, is focused as Band +II. As Band +II AFP is, on the basis of our previous studies, specific for HCC, our findings suggest that aside from fucosylated monosialo biantennary complex-type *N*-glycan (Yamashita et al, 1993; Shimizu et al, 1996), non-fucosylated monosialo biantennary complex-type *N*-glycan is also the predominant glycan moiety of the HCC-specific AFP glycoforms. The AFP glycoform carrying a single difucosylated disialo biantennary complex-type *N*-glycan, which had been identified in a hepatoblastoma cell culture (HepG2) by ESI-MS (Ferranti et al, 1995), was not found in our patient sera. Further investigation is necessary before we can answer whether hyper-fucosylation of AFP is a unique property of hepatoblastoma, but not to HCC, or whether it is due to change in cellular properties upon in vitro cell culture.

Among the predominant AFP isoforms, in our IEF system, the one that is commonly identified together with Band +II AFP in sera of HCC patients and usually occurs alone in sera of patients with benign liver disease is Band +I AFP (Ho et al, 1996; Johnson et al, 1997). This ‘benign’ isoform appears to be composed of one disialylated biantennary complex-type *N*-glycan. As fucosylation of the *N*-linked glycan has been postulated to happen only during oncogenesis because of loss of cell polarity (Yamashita et al, 1993), the non-fucosylated disialo biantennary complex-type *N*-glycan is likely to be the main carbohydrate structure of this ‘benign’ AFP. Unfortunately, the amount of serum AFP in patients with liver cirrhosis is always too low (< 500 ng mg$^{-1}$) to permit purification for the purpose of structural analysis. With the currently available technology it is therefore difficult to confirm our suggested structure for the ‘benign’ AFP isoform.

It has been reported that detection of lentil lectin-reactive AFP by lectin affinity electrophoresis may only discriminate benign liver diseases from HCC with a maximum diameter of nodules > 20 mm, but not from early malignancy (maximum diameter of nodules <10 mm) (Taketa et al, 1993). In contrast, our preliminary studies have suggested that detection of Band +II AFP may allow early diagnosis of HCC in high-risk patients even before it can be detected by routine ultrasound examination (Johnson et al, 1997). The detection of lentil lectin-reactive AFP may thus be less sensitive than Band +II AFP for early diagnosis of HCC. The appearance of monosialo-AFP (Band +II AFP) in the blood during the development of HCC in patients with chronic liver disease or cirrhosis may, in part, be related to a decrease in the sialylation activity in HCC cells. As a result, the apparent higher specificity of monosialo-AFP suggests that the decrease in the sialylation activity may occur earlier than the increase in the fucosylation activity during oncogenesis in human liver.

In conclusion, three classes of AFPs, +I, +II and +III, are identified in patient sera by ESI-MS. The predominant AFP glycoform

*© 2000 Cancer Research Campaign*

in each class carries one disialo, monosialo, or asialo biantennary complex-type *N*-glycans, respectively. These three classes correspond to 'benign AFP' (appearing as Band +I), 'HCC-specific AFP' (Band +II) and 'NSGCT-specific AFP' (Band +III), respectively. Knowledge of the structure of the tumour-specific isoforms should form the basis for clinically useful assays.

### ACKNOWLEDGEMENTS

This work was supported by a grant from the Hong Kong Research Grants Council (CUHK 255/96M). We thank Brian S Cheng for skillful technical assistance on ESI-MS. We are also grateful to the Providence Foundation Ltd, Hong Kong and the Hong Kong Cancer Fund for their continuing support of liver cancer research.

### REFERENCES

Aoyagi Y, Suzuki Y, Igarashi K, Saitoh A, Oguro M, Yokota T, Mori S, Suda T, Isemura M and Asakura H (1993) Carbohydrate structures of human α-fetoprotein of patients with hepatocellular carcinoma: presence of fucosylated and non-fucosylated triantennary glycans. *Br J Cancer* **67**: 486–492

Buamah PK, Harris R, James OFW and Skillen AW (1986) Lentil-lectin-reactive alpha-fetoprotein in the differential diagnosis of benign and malignant liver disease. *Clin Chem* **32**: 2083–2084

Burditt LJ, Johnson MM, Johnson PJ and Williams R (1994) Detection of hepatocellular carcinoma-specific alpha-fetoprotein by isoelectric focusing. *Cancer* **74**: 25–29

Dell A, Khoo K-H, Panico M, McDowell RA, Etienne AT, Reason AJ and Morris HR (1993) FAB-MS and ES-MS of glycoproteins. In: *Glycobiology, a practical approach*, Fukuda M, Kobata A (eds) pp 187–222. IRL Press: Oxford

Du MQ, Hutchinson WL, Johnson PJ and Williams R (1991) Differential alpha-fetoprotein lectin binding in hepatocellular carcinoma. *Cancer* **67**: 476–480

Ferranti P, Pucci P, Marino G, Fiume I, Terrana B, Ceccarini C and Malorni A (1995) Human α-fetoprotein produced from Hep G2 cell line: structure and heterogeneity of the oligosaccharide moiety. *J Mass Spectr* **30**: 632–638

Ho S, Cheng P, Chan A, Leung N, Yeo W, Leung T, Lau WY, Li AKC and Johnson PJ (1996) Isoelectric focusing of alpha-fetoprotein in patients with hepatocellular carcinoma – frequency of specific banding patterns at non-diagnostic serum levels. *Br J Cancer* **73**: 985–988

Javadpour N (1980) The role of biologic tumour markers in testicular cancer. *Cancer* **45**: 1755–1761

Johnson PJ and Williams R (1987) Cirrhosis and the aetiology of hepatocellular carcinoma. *J Hepatol* **4**: 140–147

Johnson PJ, Portmann B and Williams R (1978) Alpha-fetoprotein concentration measured by radioimmunoassay in the diagnosing and excluding of hepatocellular carcinoma. *BMJ* **2**: 661–663

Johnson PJ, Ho S, Cheng P, Chan A, Leung T and Yuen J (1995) Germ cell tumours express a specific alpha-fetoprotein variant detectable by isoelectric focusing. *Cancer* **75**: 1663–1668

Johnson PJ, Leung N, Cheng P, Welby C, Leung T, Lau WY and Ho S (1997) 'Hepatoma-specific' alpha-fetoprotein may permit preclinical diagnosis of malignant change in patients with chronic liver disease. *Br J Cancer* **75**: 236–240

Johnson PJ, Poon TCW, Hjelm NM, Ho CS, Ho SKW, Welby C, Stevenson D, Patel T, Parekh R and Townsend RR (1999) Glycan composition of serum alpha-fetoprotein in patients with hepatocellular carcinoma and non-seminomatous germ cell tumour. *Br J Cancer* **81**: 1188–1195

Kay PH, Wells FC and Goldstraw P (1987) A multi-disciplinary approach to primary nonseminomatous germ cell tumours of the mediastinum. *Ann Thorac Surg* **44**: 578–582

Kew MC and Popper H (1984) Relationship between hepatocellular carcinoma and cirrhosis. *Sem Liver Dis* **4**: 136–146

Lange PH and Fralay EE (1977). Serum AFP and HCG in the treatment of patients with testicular tumours. *Urol Clin North Am* **4**: 393–406

Lok ASF and Lai CL (1989) Alpha-fetoprotein monitoring in Chinese patients with chronic hepatitis B virus infection: role in the early detection of hepatocellular carcinoma. *Hepatology* **9**: 110–115

Nichols CR, Saxman S, Williams SD, Loehrer PJ, Miller ME, Wright C and Einhorn LH (1990) Primary mediastinal nonseminomatous germ cell tumours. A modern single institution experience. *Cancer* **65**: 1641–1646

Nomura F, Ohnishi K and Tanabe Y (1985) Clinical features and prognosis of hepatocellular carcinoma with reference to serum alpha-fetoprotein levels. *Cancer* **64**: 1700–1707

Okuda K (1986) Early recognition of hepatocellular carcinoma. *Hepatology* **6**: 729–738

Ruoslahti E (1979) α-fetoprotein in cancer and fetal development. *Adv Cancer Res* **29**: 275–346

Sheu JC, Sung JL, Chen DS, Yang PM, Lai MY, Lee CS, Hsu HC, Chuang CN, Yang PC, Wang TH (1985) Growth rate of asymptomatic hepatocellular carcinoma and its clinical implications. *Gastroenterol* **89**: 259–266

Shimizu K, Katoh H, Yamashita F, Tanaka M, Tanikawa K, Taketa K, Satomura S and Matsuura S (1996) Comparison of carbohydrate structures of serum α-fetoprotein by sequential glycosidase digestion and lectin affinity electrophoresis. *Clin Chim Acta* **254**: 23–40

Smith CJP and Kelleher PC (1980) Alpha-fetoprotein molecular heterogeneity: physiologic correlations with normal growth, carcinogenesis and tumour growth. *Biochim Biophys Acta* **605**: 1–31

Stibler H (1991) Carbohydrate-deficient transferrin in serum: a new marker of potentially harmful alcohol consumption reviewed. *Clin Chem* **37**: 2029–2037

Taketa K, Endo Y, Sekiya C, Tanikawa K, Koji T, Satomura S, Matsuua S, Kawai T and Hirai H (1993) A collaborative study for the elevation of lectin-reactive alpha-fetoproteins in early detection of hepatocellular carcinoma. *Cancer Res* **53**: 5419–5423

Tarelli E, Ashcroft AE, Calam DH, Corran PH, Green BN and Wheeler SF (1992) Human alpha-fetoprotein. Molecular weight data from electrospray mass spectrometry (ESMS) analysis. *Biol Mass Spectr* **23**: 315–317

Yamashita K, Taketa K, Nishi S, Fukushima K and Takashi O (1993) Sugar chains of human cord serum α-fetoprotein: characteristics of N-linked sugar chains of glycoproteins produced in human liver and hepatocellular carcinomas. *Cancer Res* **53**: 2970–2975

Yoshima H, Mizuochi T, Ishii M and Kobata A (1980) Structure of the asparagine-linked sugar chains of α-fetoprotein purified from human ascites fluid. *Cancer Res* **40**: 4276–4281

*© 2000 Cancer Research Campaign*
